# The Age of Decay

**Published:** 1891-09

**Authors:** 


### THE AGE OF DECAY.

Birth, growth, maturity, decay, death—such is the normal history of man. The three periods of life should sustain a certain proportion to each other: twenty years of growth, sixty years of maturity, twenty years of decay. This is what might be counted upon as the ordinary course of human life, but for the fact that we labor under a load of ancestral transgressions of physical and moral law, supplemented and intensified by our own personal delinquencies and follies.

How pleasant is the picture! Twenty years of happy childhood and youth, sixty years of intellectual progress and achievement, with domestic and social joys, and then twenty years of slow, almost unconscious decay, characterized by serenity of mind, pleasing memories, and joyous anticipations of a grander life beyond the grave.

Sadly different is human existence as we see it. We look with wonder upon Gladstone, past eighty, still vigorous in body and mind, still strong and wise to lead the great Liberal party of England. We accept threescore and ten as life's natural limit, and expect only labor and sorrow if this limit is passed.

We are doomed, we think, by our inheritance; and to some extent this is true. But we should remember the law of recuperation. The torn flesh heals; the broken bone reunites. Diseases tend toward recovery. The weary toiler rises from sleep strong for new labors. The wise physician bases his hopes upon this law.

And this tendency of nature to heal herself may be greatly assisted by careful and intelligent living, so that it is always possible that the man of unfortunate ancestry may secure for himself a good old age, and start his posterity upon an ascending plane.

Do what we will, however, life must have its end. When the age of decay is reached, hidden changes are going on, the culmination of which is the last great change. The muscles shrink; the brain shrivels; nerves lose their sensibility and active power; the arteries, perhaps, become chalky or fatty; the heart is weakened; the circulation enfeebled; and at last the end comes.

During this final period, then, we must take things calmly; avoid excesses of all kinds; guard against exposures to cold; keep up a degree of mental activity; cultivate cheerfulness, and look forward with hope.—*Ex.*

